# Vehicle-Mounted Solar Occultation Flux Fourier Transform Infrared Spectrometer and Its Remote Sensing Application

**DOI:** 10.3390/s23094317

**Published:** 2023-04-27

**Authors:** Yasong Deng, Liang Xu, Xianchun Sheng, Yongfeng Sun, Hanyang Xu, Huanyao Xu, Haotian Wu

**Affiliations:** 1 Key Laboratory of Environmental Optics and Technology, Anhui Institute of Optics and Fine Mechanics, Chinese Academy of Sciences, Hefei 230031, China; ysdeng@aiofm.ac.cn (Y.D.);; 2 Science Island Branch of Graduate School, University of Science and Technology of China, Hefei 230026, China

**Keywords:** Fourier transform infrared spectrum, solar occultation flux, solar tracker, remote sensing application

## Abstract

For the demand of rapid monitoring of pollution gas disorganized emissions in industrial parks, this paper studies the solar fast tracker system of vehicle-mounted SOF-FTIR (Solar Occultation Flux Fourier Transform Infrared Spectroscopy) system, where the spectrometer directly measures the broadband absorption spectrum of solar radiation light. A fast portable solar tracking system based on PSD (position sensitive detector) is designed, the mathematical model of solar spot position on the PSD surface source is established, and the optimal optical design parameters are simulated using the model. The dead-zone integral separation PID (Proportion Integration Differentiation) control algorithm is used to track the trajectory of the solar, and the light spot position model is used to nonlinearly compensate the output of PID control so that the PID controller has the same control precision and response speed in different error areas. Experimental analysis of the solar tracking performance of the vehicle-mounted SOF-FTIR under static and dynamic conditions, as well as the spectral effects on the measurements under static vehicle, constant speed, and turning driving conditions. The remote sensing application experiment of vehicle-mounted SOF-FTIR pollution gas emission flux was carried out in a tire factory in Hefei City, Anhui Province. A vehicle-mounted SOF-FTIR system realized the qualitative and quantitative analysis of the pollution gas at the boundary of the tire plant and calculated the flux of each component pollution gas. The emission flux of pollution gas was highly consistent with the actual pollution distribution of the tire plant. The results show that the positioning accuracy of PSD in the vehicle tracking experiment can also meet SOF-FTIR requirements for solar tracking. The remote sensing system will be useful in the field of atmospheric environment monitoring, and the mobile monitoring of regional pollutant gases based on solar infrared spectroscopy has application value.

## 1. Introduction

VOCs (volatile organic compounds) generated in industrial processes are potentially harmful to human health and the environment due to unorganized emissions [1]. Therefore, it is important to find a means to monitor the concentration column of pollutant gases in the atmosphere. With wind speed and direction parameters, SOF-FTIR (Solar Occultation Flux Fourier Transform Infrared Spectroscopy) was used to invert the gas column concentration based on the broad-band absorption spectrum of solar infrared radiation [2]. Johan Mellqvist et al. observed industrial olefin emissions in Texas using the solar occultation flux technique [3]. Sunil Baidar et al. combined the imaging device with a motion compensation system to achieve a high level of angular accuracy, and the time required to find the sun in the sky was eliminated, thus improving the duty cycle of the instrument, but a rough manual alignment was required to position the tracker reflector and the motion compensation system [2].

Donato Vincenzi et al. used a high-speed camera to acquire solar images during tracking and calculate the declination of the structure concerning the reference direction angle [4]. Based on computer vision and photoelectric sensors, R. Ahmed et al. built an active day-lighting dual-axis sun tracking system capable of identifying objects (such as the sun or clouds) captured by the camera and processing the data required by the solar tracker [5]. Diego Rativa et al. designed a four-quadrant sensor based on fiber coupling and studied its application in solar tracking in different orientations [6]. However, the image acquired by the above image processing method under the weather condition of weak illumination is fuzzy, which cannot meet SOF-FTIR requirements for high-precision tracking. Compared with the PSD sensor method, the scanning speed of machine vision and the fiber-coupled four-quadrant sensor method is slow, the processing algorithm is complex, and it is difficult to meet the demand of SOF-FTIR for the fast movement tracking of the solar in practical applications. The tracking on the moving platform can be compensated to some extent by using astronomical positioning or increasing motion compensation, but it undoubtedly increases the complexity of the system and reduces the reliability of the system operating in field conditions. In previous research on a solar tracking system based on PSD, researchers regarded the trajectory of light spots on the PSD in the two-dimensional scanning process as an orthogonal straight line, and the orthogonal coordinate system remained unchanged with the change in the solar altitude angle. However, there is a nonlinear relationship between rotation angle and spot displacement, and the actual trajectory motion is not a straight trajectory with an orthogonal position.

Compared with the image detection method, the PSD method has the advantages of fast response time, good positioning accuracy, and a simple signal conditioning circuit because it has a position resolution of a micron and a time resolution of a microsecond. After analyzing the influence of various factors on PSD measurements, David R. N. proposed an electrical calibration method based on PSD [7]. PSD has been widely used in micro/nano position measurements, vibration detection, tool alignment, aiming and guidance systems, and position detection by direct irradiation of a laser or another light source on the PSD surface [8]. Based on the application of PSD in the solar spectrum measurement system, Qu Liguo et al. designed a PSD signal processing circuit and proposed a static voltage inference calibration method based on a genetic algorithm that can achieve a positioning accuracy of 0.01 mm when the light intensity is weak [9]. 

Vehicle-mounted SOF-FTIR, due to the vehicle itself bumps, car turns, buildings, or shade obstacles that block the PSD position, is easily lost, affecting the spectral quality and gas concentration inversion accuracy. SOF-FTIR is needed to accurately and quickly track the sun during the movement. Jin Ling et al. adopted the method of deviating position hierarchical tracking model for fast tracking, but it needed 100 ms to complete a solar position correction, which could not meet the requirements of fast tracking in the motion process [10]. The purpose of adding integral control to the process of ordinary PID motion control is to eliminate static error and improve control accuracy, but it will cause integral accumulation of PID operation and cause large overshoot.

This paper researches a solar tracking system based on PSD to solve the above problems and successfully applies it to SOF-FTIR. First, the system composition of SOF-FTIR and the hardware design of the sun-tracking system are introduced; a trajectory model of the sunspot on PSD is established; and a solar tracking algorithm based on integral separation PID control with the dead zone is proposed. In the second two tests, the performance of the sun tracking system is tested under static and dynamic conditions, the SOF-FTIR tracking application experiments are studied, and the pollutant gas fluxes in each area, The results are in Section 3. Finally, some conclusions are given in the conclusion section.

## 2. SOF-FTIR System Design and Research

**Figure 1:**
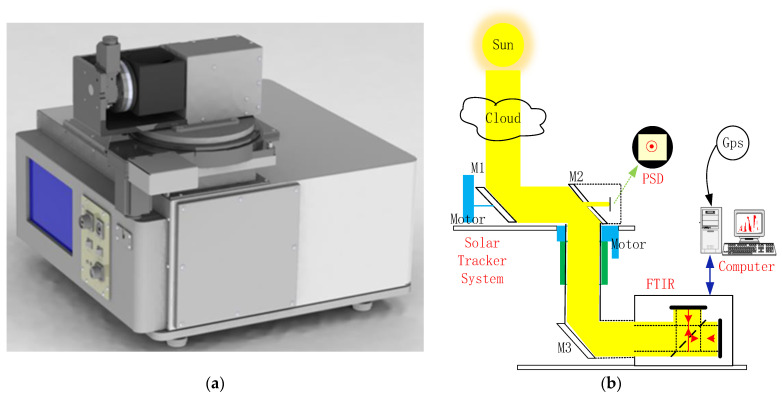
The combination of the SOF-FTIR system: (**a**) SOF-FTIR system model and (**b**) SOF-FTIR system structure diagram.

The SOF-FTIR system (as shown in Figure 1a) consists of the optical system, electronics module, GPS (Global Positioning System) module, mechanical system, FTIR (Fourier Transform Infrared Spectroscopy) spectrometer, PC (Personal Computer), and user interface software. The optical system, electronics module, GPS, and mechanical system constitute the solar tracking system (as shown in Figure 1b). The precision of solar tracking directly affects the signal quality of the spectrum [11]. In the SOF-FTIR system, altitude angles and azimuths were calculated based on longitude and latitude information, and real-time solar azimuths were calculated in combination with gyroscope parameters. PSD was used to record the changes in the sun’s position accurately. 

### 2.1. FTIR System Design

**Figure 2:**
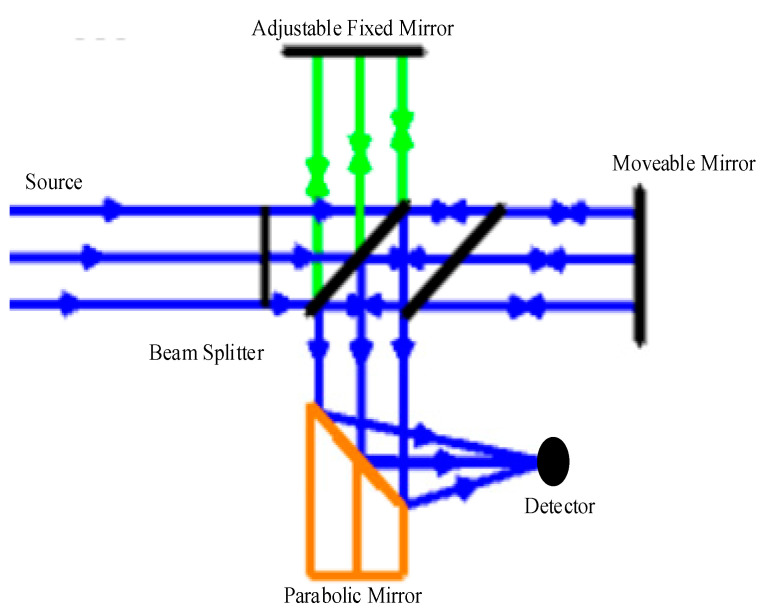
Schematic diagram of a dynamic collimated interferometer.

A Michelson interferometer is the core of the Fourier transform infrared spectroscopy system. Interferograms are Fourier transformed to obtain infrared absorption spectra for the qualitative and quantitative analysis of various substances. Figure 2 shows the simplest structure of the Michelson interferometer optical path, which consists of two mutually perpendicular mirrors, one of which can be moved along an axis perpendicular to the other mirror (fixed mirror). Between the two mirrors is the beam splitter. The infrared light is divided into two perpendicular beams through the beam splitter. The upwardly reflected light is reflected on the fixed mirror and then converged on the detector by the objective lens. Another beam of light is reflected by a moving mirror and then concentrated on the detector by a parabolic mirror. Two light waves with an optical path difference interfere on the detector, and the total light intensity depends on the optical path difference of the two beams. 

### 2.2. Solar Tracking Trajectory Modeling 

**Figure 3:**
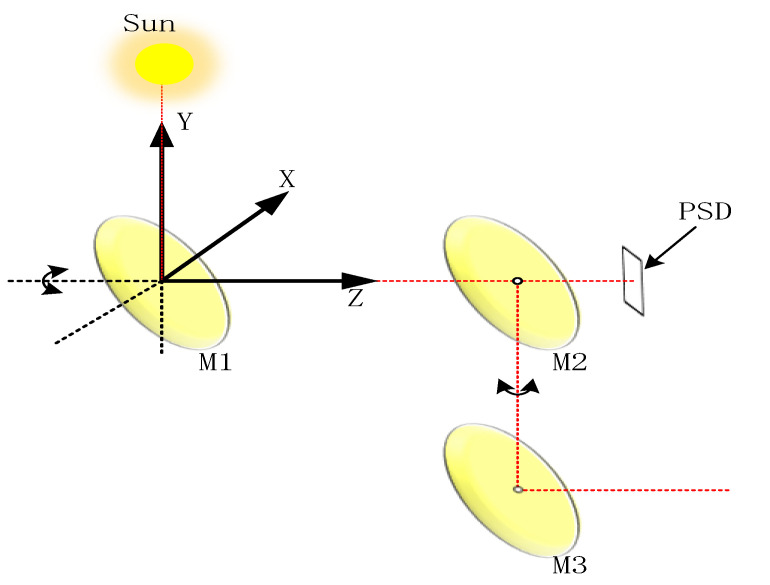
The position coordinate system of the solar and the spot trajectory on the PSD.

According to the position relation between the sun and the spot track on PSD in the SOF-FTIR system (as shown in Figure 3), a three-dimensional coordinate system is established: the center of the first mirror M1 is the origin, the central axis of the first mirror M1 and the second mirror M2 is the *Z* axis, the vertical paper inward direction is the *X* axis, and the vertically upward direction is the *Y* axis.

**Figure 4:**
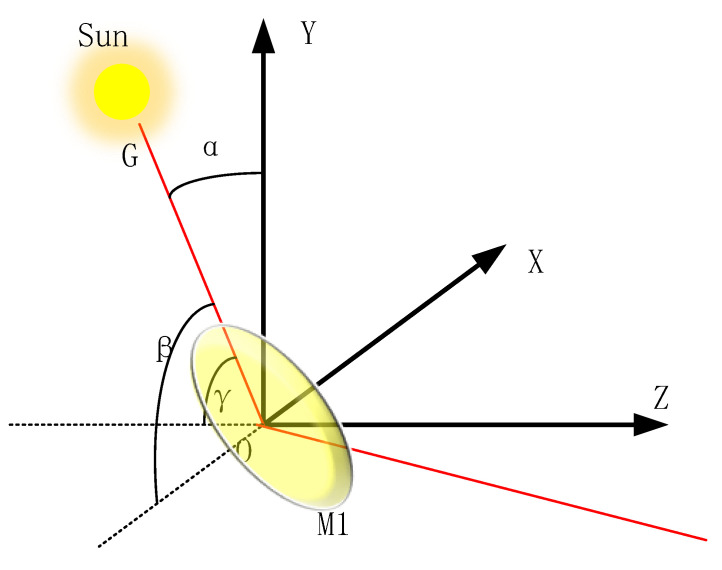
Schematic diagram of M1 spatial location coordinates.

The parallel light from the solar is equated to a thin beam passing through the center of mirror M1. When a certain angle arbitrarily rotates the first mirror M1, it is equivalent to the center normal *N* of M1 rotating *β* around the *X*-axis, *α* around the *Y*-axis, and *γ* around the *Z*-axis in the *X-Y-Z* three-dimensional coordinates. Assuming that the solar position remains unchanged, the trajectory of the spot at one end of the PSD is analyzed with the rotation of the sweeping mirror (as shown in Figure 4). If the solar incident light *G* is in the X-O-Z dimension and the angle with the *X*-axis is *δ*.

When the solar altitude angle is 0°, which is equivalent to the first reflector M1 rotated along the *Z*-axis at an angle *θ* = 0, the solar incident light unit vector is *N* (1, 0, 0) and the normal vector is $N_{0}=\left|0,\frac{\sqrt{2}}{2},\frac{\sqrt{2}}{2}\right|$. When the solar incident light is rotated along the *Z*-axis at *θ*, the normal vector $N_{\theta }$ and the solar reflected light unit cosine $G_{\theta }$ are:

(1) $$N_{\theta }=\left|\frac{\sqrt{2}}{2}\cos\theta ,\frac{\sqrt{2}}{2}\sin\theta ,\frac{\sqrt{2}}{2}\right|$$

$$G_{\theta }=(\cos\beta ,\tan\alpha ,-\sin\beta )$$

The expression of the reflection action matrix $R_{\theta }$ is calculated in Equation (1):

(2) $$R_{\theta }=\left|\begin{array}{ccc} \sin^{2}\theta & -\cos\theta •\sin\theta & -\cos\theta \\ -\cos\theta •\sin\theta & \cos^{2}\theta & -\sin\theta \\ -\cos\theta & -\sin\theta & 0 \end{array}\right|$$

The vector of reflected rays from the solar is:

(3) $$\left[\begin{array}{l} G_{x}\\ G_{y}\\ G_{z} \end{array}\right]=\left[\begin{array}{ccc} \sin^{2}\theta & -\cos\theta •\sin\theta & -\cos\theta \\ -\cos\theta •\sin\theta & \cos^{2}\theta & -\sin\theta \\ -\cos\theta & -\sin\theta & 0 \end{array}\right]•\left[\begin{array}{l} \cos\beta \\ \tan\alpha \\ -\sin\beta \end{array}\right]$$

Let the distance from the center point O of the first reflector M1 to the center position of the PSD be *d*. Then,

(4) $$\begin{array}{l} G_{z}=d\\ G_{x}=d•\frac{-\sin^{2}\theta \cos\beta +\cos\theta \sin\theta \tan\alpha -\cos\theta \sin\beta }{\cos\theta \cos\beta +\sin\theta \tan\alpha }\\ G_{y}=d•\frac{\cos\theta \sin\theta \cos\beta -\cos^{2}\theta \tan\alpha -\sin\theta \sin\beta }{\cos\theta \cos\beta +\sin\theta \tan\alpha } \end{array}$$

The solar altitude angle is *γ*, θ=γ+α:

(5) $$\begin{array}{l} G_{z}=d\\ G_{x}=d•\frac{-\sin^{2}(\gamma +\alpha )\cos\beta +\cos(\gamma +\alpha )\sin(\gamma +\alpha )\tan\alpha -\cos(\gamma +\alpha )\sin\beta }{\cos(\gamma +\alpha )\cos\beta +\sin(\gamma +\alpha )\tan\alpha }\\ G_{y}=d•\frac{\cos(\gamma +\alpha )\sin(\gamma +\alpha )\cos\beta -\cos^{2}(\gamma +\alpha )\tan\alpha -\sin(\gamma +\alpha )\sin\beta }{\cos(\gamma +\alpha )\cos\beta +\sin(\gamma +\alpha )\tan\alpha } \end{array}$$

**Figure 5:**
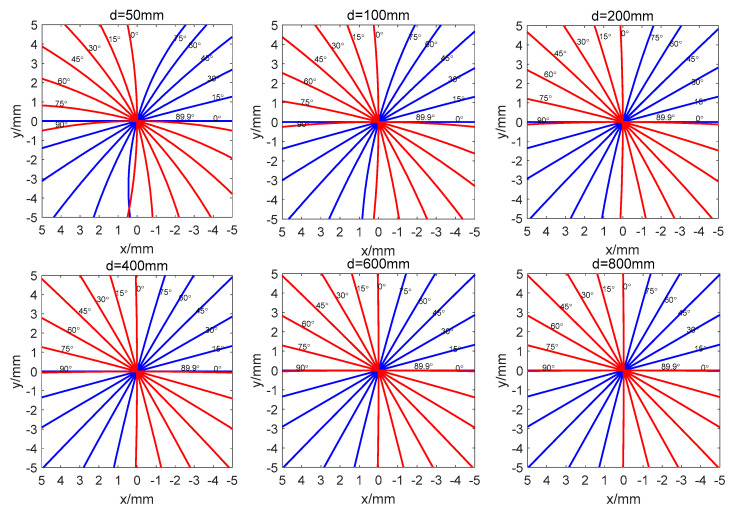
The trajectory of PSD spot movement for different *d* and different *γ* cases (blue is the *x*-axis, red is the *y*-axis).

The Matlab simulation results of the PSD spot movement trajectory under different *d* and different *γ* conditions (as shown in Figure 5). The results show that, with an increase in d, the trajectory of the light spot gradually approaches the orthogonal line in the X and Y axes. As *γ* varies between 0 and 90 degrees, the orthogonal coordinate system rotates *γ* relative to the Earth’s coordinate system. 

The PSD spot trajectory model’s simulation results based on Matlab show a nonlinear relationship between the motor rotation angle and spot displacement. The output of PID control is nonlinearly compensated with PSD’s spot trajectory model to ensure that the PID controller has the same control precision and response speed in different error areas so that the same group of PID parameters have the same control effect without error.

### 2.3. Trajectory Tracking Based on Dead-Zone Integral Separation PID Control Algorithm

When the saturation effect, hysteresis, eddy current loss, magnetic leakage between stator poles and ends, magnetic leakage of the permanent magnet loop, the self-induced harmonic component of the stator coil, and no load of the motor are ignored, the motion equation of a two-phase hybrid stepper motor can be obtained as follows:

(6) $$J\frac{d^{2}\theta }{dt}+B\frac{d\theta }{dt}-\frac{ZLi^{2}}{2}\sin(Z•\theta )=0$$

The transfer function model of the stepper motor is obtained by the Laplace transform from the following formula:

(7) $$G(s)=\frac{Li^{2}Z^{2}}{2Js^{2}+2Bs+Li^{2}Z^{2}}$$

*J* is the rotational inertia, *B* is the viscous friction coefficient, *Z* is the number of motor rotor teeth, *L* is the winding self-inductance, and I is the single-phase current.

Vehicle-mounted SOF-FTIR is a highly dynamic control system. Because of the influence of the initial tracking position, road conditions, shade trees, buildings, and other factors in the working process, the position of the tracking system needs to be adjusted quickly. To eliminate the oscillation caused by frequent movements of the control system, SOF-FTIR adopts dead-zone PID control to perform rough tracking of the solar tracking system. The PID control formula can be expressed as (8):

(8) $$error(k)=\left\{ \begin{array}{l} 0,|error(k)|\leq |e_{0}|\\ error(k),|error(k)|>|e_{0}| \end{array}\right.$$

*Error* (*k*) is the position tracking deviation; *e*_0_ is an adjustable parameter, and its value is determined according to the actual control object. If the value of *e*_0_ is too small, the system control action is too frequent, and the purpose of stabilizing the controlled object cannot be achieved. If the value of *e*_0_ is too large, the system control action will lag.

Due to the limited PSD point source, the solar tracking system will lose a light spot if there is a large overshoot after the completion of rough tracking. Therefore, integral separation PID is used to control accurate tracking and avoid an increase in overshoot. Integral separation PID control is to cancel the integral function when the deviation between the controlled quantity and the set value is large. The controlled quantity is close to the given value, and integral control is introduced to eliminate the static error and improve the control precision. According to the light intensity value under different weather conditions (the light intensity value can be obtained from the startup self-test process), set the threshold *μ*. When |*error* (*I*)| > *μ*, avoid excessive overshoot. Using PD (proportion differentiation) control gives the system a faster response. When |*error* (*I*)| ≤ *μ*, PID control is used to ensure the control precision of the system.

The integral separation PID control algorithm can be expressed as (9):

(9) $$u(I)=k_{p}error(I)+\varphi k_{i}\sum_{j=0}^{k}error(I)T+k_{d}(error(I))-error(I-1))/T$$

where *T* is the sampling tim, and *Φ* is the switching coefficient of the integration term.

(10) $$\varphi =\left\{ \begin{array}{l} 1,|error(I)|\leq \mu \\ 0,|error(I)|>\mu \end{array}\right.$$

In the formula, the value of *Φ* depends on the light intensity. If *Φ* is too large, it will not achieve the purpose of integral separation. If *Φ* is too small, it will lead to the inability to enter the integration zone; if only PD control is carried out, it will make the control appear as the residual difference.

**Figure 6:**
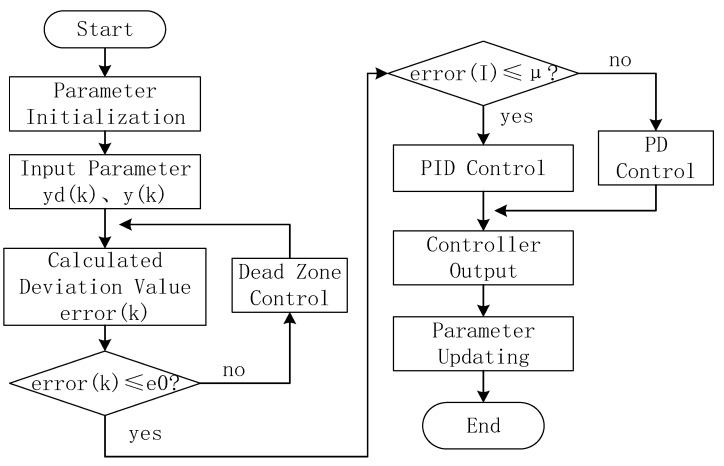
Flow chart of the dead-zone integral separation PID control.

According to the dead-zone integral separation PID control algorithm to obtain its program block diagram (as shown in Figure 6).

**Figure 7:**
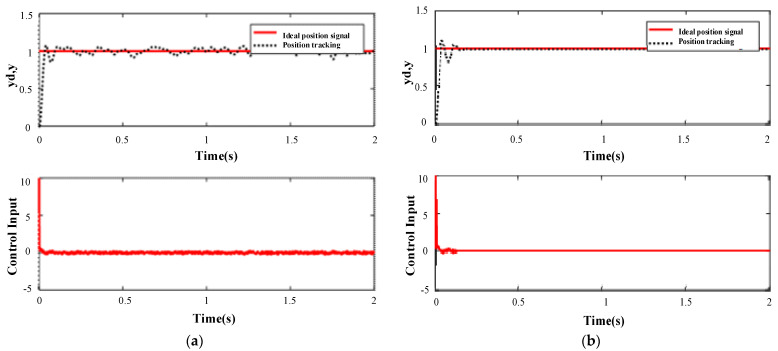
Simulation results of the PID control algorithm PID: (**a**) integral split PID control and (**b**) integral split PID control with dead zone.

PID control simulation is carried out in Matlab. The threshold *μ* is 0.2, the dead zone parameter *e*_0_ is 0.1, and the normally distributed random interference signal of 0.5 is added. The simulation results are shown in Figure 7. The general integral separation PID control method is adopted (as shown in Figure 7a). Overshoot is 0.058, steady-state error (using the steady-state interval of ±2%) is 0.02, and the time to enter the steady state is 1.86 s. The integral separation PID control method with dead zone was adopted (as shown in Figure 7b), the overshoot was 0.11, the steady-state error (using the steady-state interval of ±2%) was 0.015, and the time to enter the steady-state was 0.16 s. It can be seen from the simulation results that the output of an integrally separated PID controller with a dead zone is more stable.

Therefore, the solar tracking method of the mobile SOF-FTIR system based on the dead-zone integral separation PID control algorithm can achieve fully automatic 360° tracking control of the solar orientation, and the time to steady state is reduced by a factor of 11.6.

## 3. Vehicle SOF-FTIR System Performance Test

**Figure 8:**
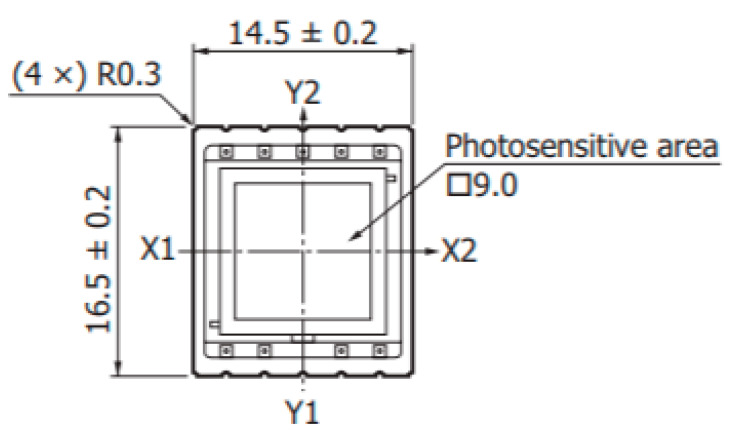
PSD Module.

The PSD (Hamamatsu S5991-01) sensor used by SOF-FTIR has a square area of 9.0 × 9.0 mm of light sensitivity (as shown in Figure 8) [12]. Based on the current obtained from the four electrodes of the PSD, we can calculate the position coordinates of the spot in the PSD.

x and y represent the position coordinates of the light spots. Let the current value in the x1 direction be I_1_, the current value in the x2 direction be I_2_, the current value in the y1 direction be I_3_, the current value in the y1 direction be I_4_, and L = 10 mm. Equation (11) is the expression for calculating the position of the solar spot.

(11) $$\begin{array}{l} \frac{\left(I_{2}+I_{3})-(I_{1}+I_{4}\right)}{I_{1}+I_{2}+I_{3}+I_{4}}=\frac{2x}{10}\\ \frac{\left(I_{2}+I_{4})-(I_{1}+I_{3}\right)}{I_{1}+I_{2}+I_{3}+I_{4}}=\frac{2y}{10} \end{array}$$

**Figure 9:**
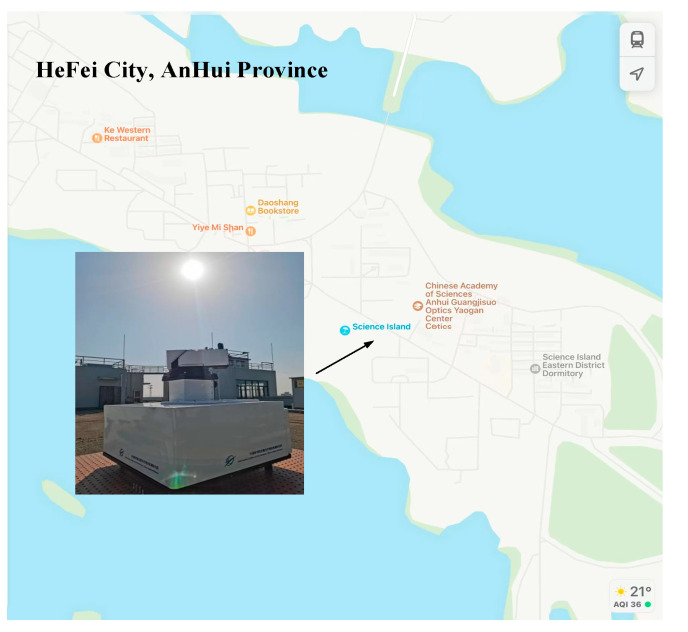
Fixed-point solar tracking experiment.

To test the performance of the system, the solar tracking effect under static conditions was first tested. The solar tracking experiment was carried out at the Science Island of Hefei City, located at 31°54′31″ N latitude, 117°9′36″ E longitude, and 40 M above sea level (as shown in Figure 9). Before the experiment started, mechanical calibration aligned the solar spot with the center point of the PSD.

**Figure 10:**
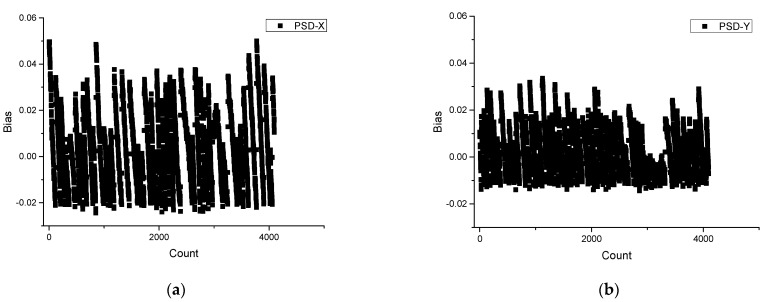
Fixed-point solar tracking spot position data: (**a**) The *X*-axis center position on the PSD is a deviation; (**b**) the *Y*-axis center position on the PSD is a deviation.

The abscissa is the number of sampling points, and the ordinate is the distance from the center of the solar spot. The experimental results show that the maximum deviation is 0.05 mm and the average deviation is 0.014 mm relative to the *X*-axis center position on the PSD in the static tracking process (as shown in Figure 10a). The maximum deviation of the center position of the *Y*-axis is 0.035 mm, and the average deviation is 0.01 mm (as shown in Figure 10b).

To analyze the spectrum of solar tracking under static conditions, the stability of continuous measurement of the system is verified by consistency analysis of the eight spectra continuously measured by SOF-FTIR (as shown in Figure 10). The spectrum showed good consistency and no obvious difference.

**Figure 11:**
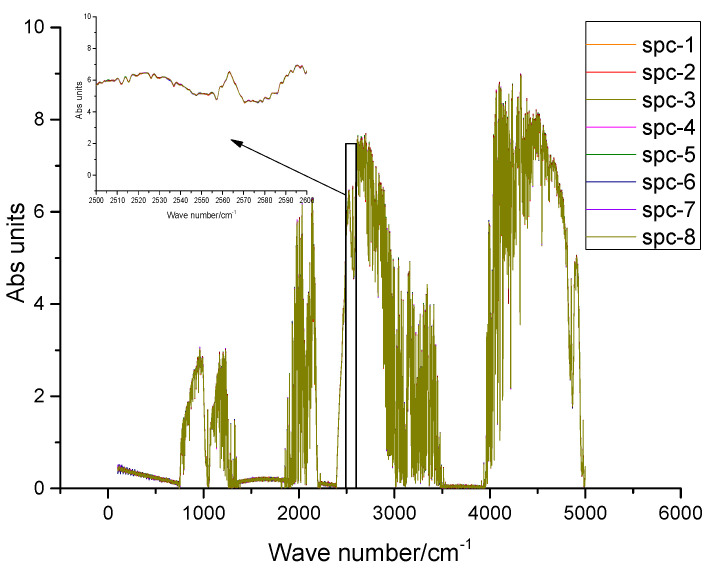
FTIR spectra under static conditions.

To analyze the influence of the solar tracking system on the quality of the FTIR spectrum, the instrument R_SNR_ (Signal-to-Noise Ratio) is usually used to evaluate the performance of infrared instruments [13]. As expressed in (12), 2500 cm^−1^~2600 cm^−1^ was selected for SOF-FTIR instrument signal-to-noise ratio analysis [14]. The calculated SOF-FTIR system R_SNR_ is 6.207 × 10^3^:1 (as shown in Figure 11), and the system R_SNR_ has no obvious change.

(12) $$R_{SNR}=\frac{100}{N}$$

*N* is the noise peak—the peak measured by the transmissivity representation.

**Figure 12:**
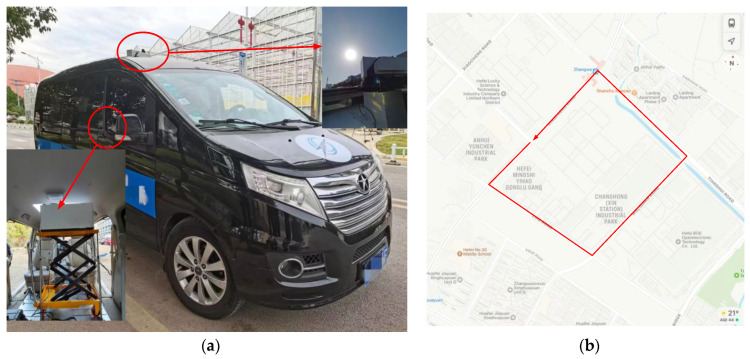
Moving solar tracking experiment: (**a**) Track the car installation drawing; (**b**) track the roadmap.

To test the reliability of solar tracking and the effect of moving solar tracking, a vehicle-mounted solar tracking experiment was carried out in Hefei City. The SOF-FTIR instrument is mounted on a JAC vehicle and extends to the top of the vehicle via a lift platform (as shown in Figure 12a). The tracking vehicle makes a circle around the factory, with an average speed of 40 km/h in a straight line and a maximum speed of 20 km/h in curves (as shown in Figure 12b).

**Figure 13:**
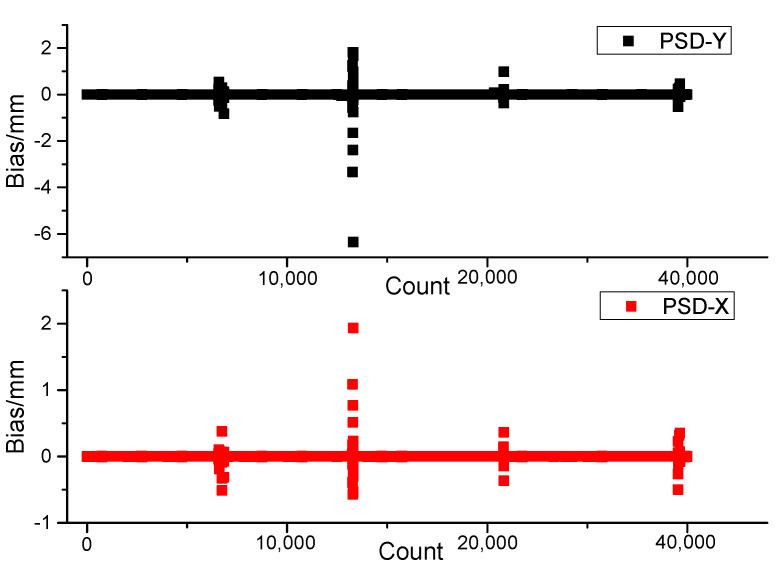
Moving solar tracking spot position data.

The four large oscillating positions are the four turning positions of the vehicles on the route (as shown in Figure 13). The experimental results show that the spot position is always in the sensitive region of the PSD at a speed of 40 km/h. When driving in a straight line, the maximum deviation of PSD position data and the average deviation of the PSD center position in the sun tracking process are 0.4 mm and 0.2 mm, respectively.

**Figure 14:**
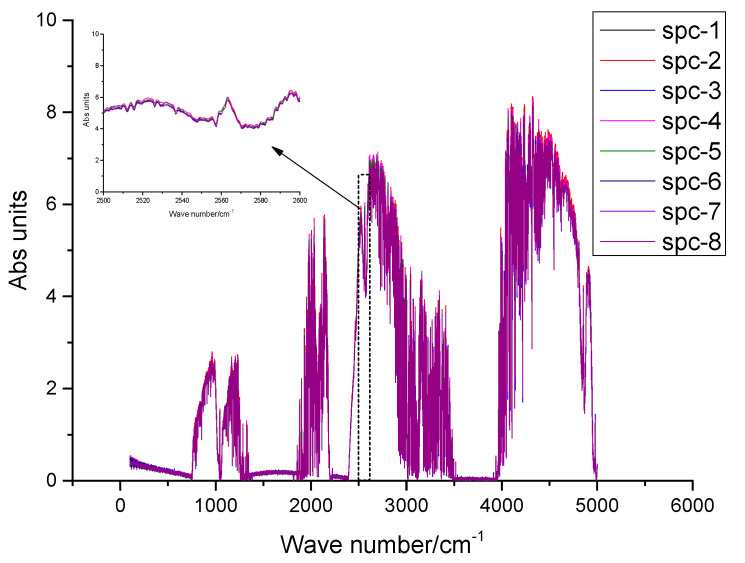
FTIR spectra under straight-line driving conditions.

To analyze the spectrum of solar tracking under the condition of straight-line travel, The stability of continuous measurement of the system is verified by consistency analysis of the eight spectra continuously measured by SOF-FTIR (as shown in Figure 14). The spectral intensity fluctuates slightly, but the spectrum shows good consistency. The calculated R_SNR_ of the SOF-FTIR system is 5.703 × 10^3^:1, and the R_SNR_ of the SOF-FTIR system is reduced by 8.2% compared with the static condition.

**Figure 15:**
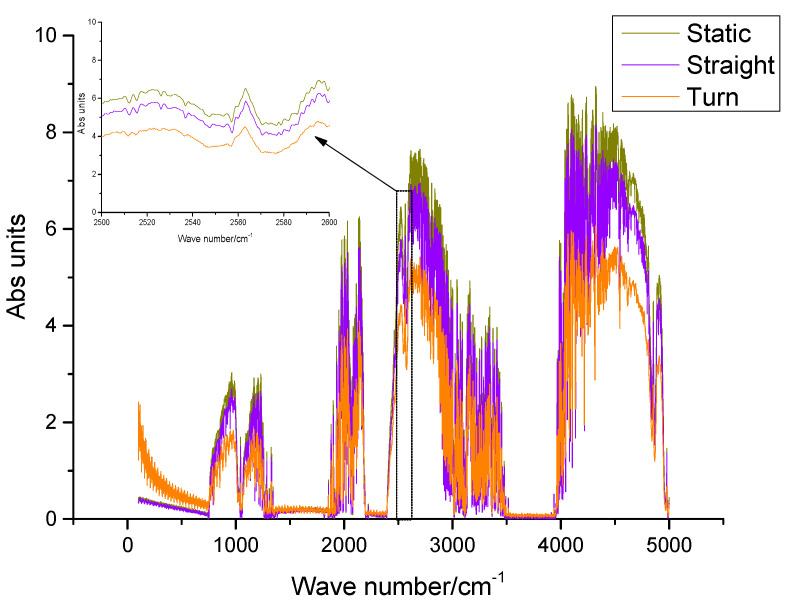
Comparison spectra of solar tracking under static, straight, and turning driving conditions.

To analyze the spectrum of solar tracking under turning driving conditions, the three spectra collected under the conditions of static, linear driving, and maximum turning angle were compared and analyzed (as shown in Figure 15). It is calculated that the SOF-FTIR instrument R_SNR_ under turning driving conditions is 3.448 × 10^3^:1, the SOF-FTIR instrument R_SNR_ is reduced by 44.5% compared with the static condition, the spectral intensity is reduced by 39.6% compared with the static condition, and the weak absorption peak is weakened. Therefore, the stability of the moving mirror of the spectrometer may be affected under the condition of turning. Multiple measurements can be made to take the average spectrum to improve the R_SNR_. The higher the average number, the stronger the noise suppression ability, which can reduce the stability requirements of the moving mirror.

**Figure 16:**
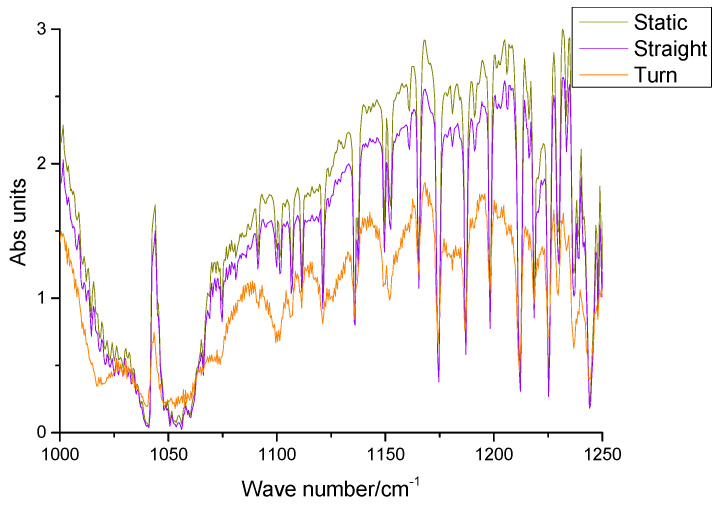
No target gas absorption FTIR spectra from 1000 cm^−1^ to 1250 cm^−1^.

To analyze the factors affecting spectral quality under three conditions, the 1000 cm^−1^~1250 cm^−1^ band without target gas absorption was selected (as shown in Figure 16). That the absorption profile of the spectral line becomes weak and the spectral resolution decreases under the turning condition. Simultaneously, the spectral line also has high-frequency oscillation, which reduces the R_SNR_ of the SOF-FTIR instrument, restrains the detection limit when the method measures gas, and brings some errors to the inversion results of column concentration. Therefore, it is an effective method to delete the spectrum of turning measurements according to spectral intensity during moving measurements to ensure the accuracy of column concentration inversion.

## 4. Results and Analysis of Solar Spectral Remote Sensing Applications

**Figure 17:**
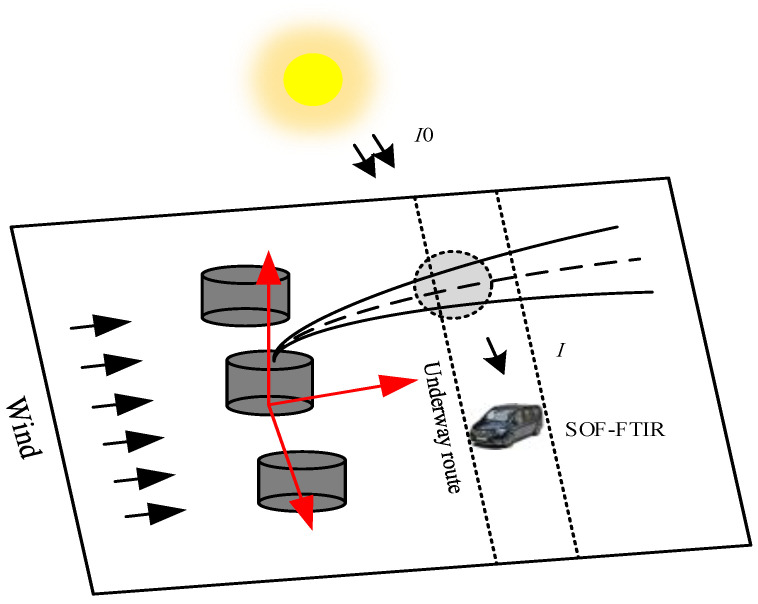
SOF-FTIR gas absorption schematic.

During SOF-FTIR operation, sunlight passes through contaminated gas and enters the spectrometer through the sun tracking system and forward optical path (as shown in Figure 17). The standard absorption cross-section of pollutant molecules was extracted from the authoritative database, and the nonlinear least square fitting method based on Levenberg–Marquardt was used to invert the column concentration of pollution components at each sampling point of the plume profile. Based on the column concentration, wind speed, wind direction, and GPS, the Gauss model calculates this area’s vertical flux of pollution gas. See Table 1 for the main technical indicators of SOF-FTIR.

**Figure 18:**
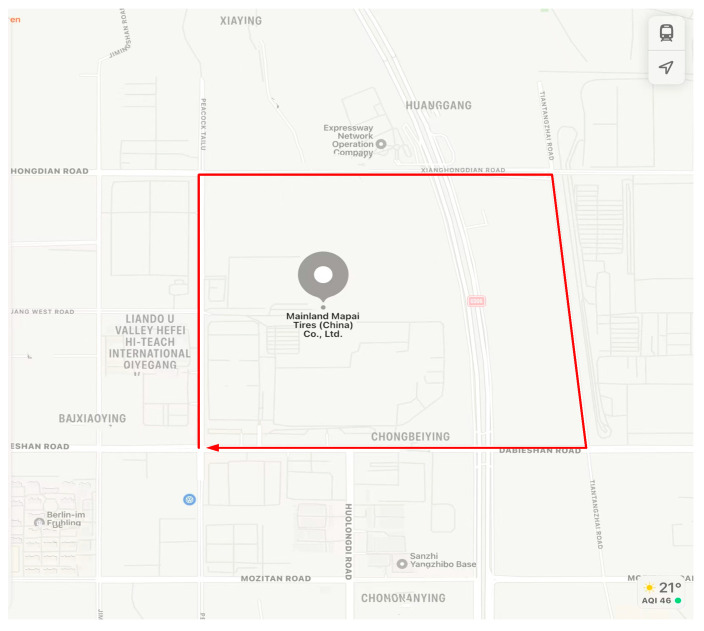
Monitoring experiment in a tire industrial park in Hefei.

To verify the practical application effect of the SOF-FTIR system, an application experiment of on-board SOF-FTIR pollution gas emission flux was carried out in a tire industrial park in Hefei City (as shown in Figure 18). The tracking car drives around the perimeter of the factory with a straight-line speed of 40 km/h and a curved speed of 20 km/h. The total distance of one lap was about 4.4 km.

**Figure 19:**
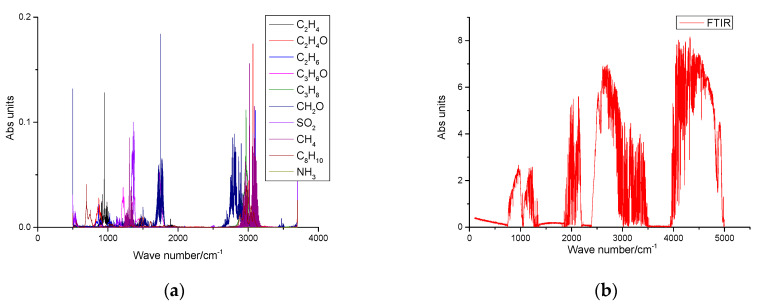
(**a**) Characteristic spectrum of the gas to be measured and (**b**) measured spectrum.

The production process of rubber tires includes raw rubber mixing, tire ring manufacturing, tire forming, tire vulcanization, and other processes. The pollution gases that may be produced in the production process include Methane (CH_4_), Formaldehyde (CH_2_O), Acetaldehyde (C_2_H_4_O), Acetone (C_3_H_6_O), Sulfur Dioxide (SO_2_), Ethylene (C_2_H_4_), Ethane (C_2_H_6_), Propane (C_3_H_8_), Paraxylene (C_8_H_10_), and Ammonia (NH_3_). The absorption cross sections of the gas components to be analyzed were selected from the HITRAN database and the QAsoft database. The absorption characteristics and measured spectra of the components to be measured are shown in Figure 19a,b.

**Figure 20:**
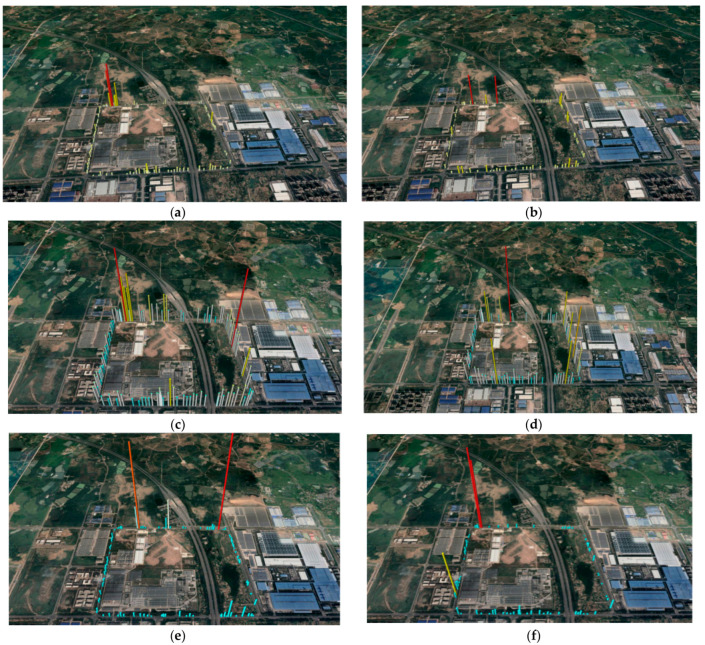
The concentration distribution of each component at different speeds: (**a**) C_2_H_4_ measurement results at 20 km/h; (**b**) C_2_H_4_ measurement results at 40 km/h; (**c**) C_3_H_8_ measurement results at 20 km/h; (**d**) C_3_H_8_ measurement results at 40 km/h; (**e**) NH_3_ measurement results at 20 km/h; and (**f**) NH_3_ measurement results at 40 km/h.

SOF-FTIR cycles around the boundary of the industrial park for spectrum acquisition, continuous measurement of two laps, and average speed control of 40 km/h, 20 km/h, wind direction southwest, and wind speed 2.29 m/s. After the quantitative analysis of the spectrum collected from the tire industrial park, the experimental results show that the main pollutants in the industrial park are C_2_H_4_, C_3_H_8_, and NH_3._ The maximum column concentration values for C_2_H_4_, C_3_H_8_, and NH_3_ are different, and different height factors are used for the concentrations shown in Google Earth (as shown in Figure 20).

**Figure 21:**
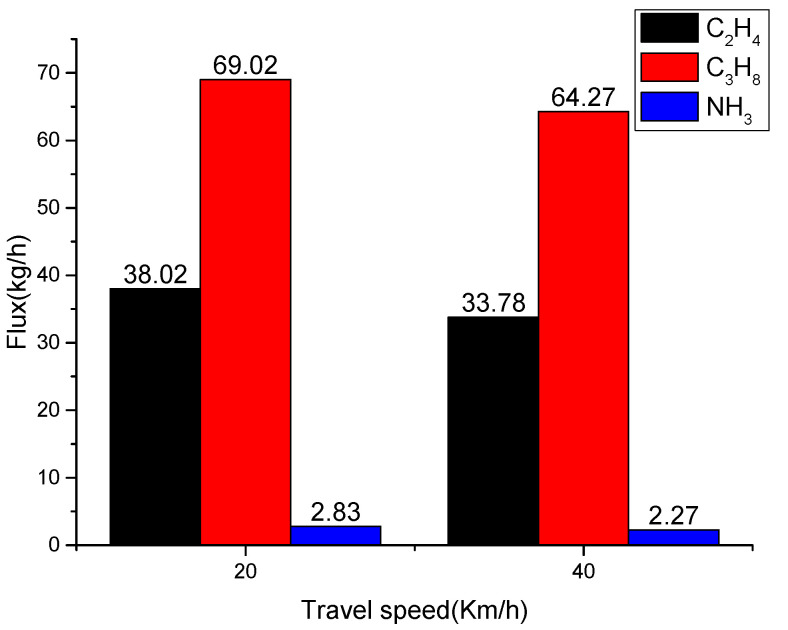
Calculation results of pollutant gas fluxes in each region.

Combined with the map and the site environment, rubber tires’ main production area is concentrated west of the industrial park. The results of two measurements show that the discharge area of the major pollutants C_2_H_4_, C_3_H_8_, and NH_3_ in the industrial park is concentrated in the northwest corner. The C2H4 column concentration reached a maximum of 203.75 mg/m^2^; the C_3_H_8_ column concentration reached a maximum of 181.46 mg/m^2^; and the NH_3_ column concentration reached a maximum of 30.15 mg/m^2^, which is consistent with the site environment. The column concentration distribution of each component changes with the wind direction, and the position of the maximum column concentration also changes. Simultaneously, the position of the maximum column concentration differs from one component to another. Combined with the calculation formula of the wind speed, direction, and flux, the pollution gas flux of each component in the plant area was calculated. The emission flux of C_2_H_4_, C_3_H_8_, and NH_3_ in the industrial park is relatively consistent (as shown in Figure 21).

The experimental results show that the SOF-FTIR system can perform qualitative and quantitative analysis of the pollution gas and invert the distribution of the pollution gas concentration. Simultaneously, combined with the calculation method of pollution gas flux, the pollution gas flux of each component is calculated.

## 5. Conclusions

The fast solar tracker system was applied to vehicle-mounted FTIR spectrometer. Compared with previous similar instruments, the 360° automatic closed-loop tracking of the sun position is achieved according to the position change of the sun spot in the PSD without a rough manual calibration to position the tracking reflector and the motion compensation system, while the system lifts the restriction on vehicle speed. The average deviation of the PSD spot position under static conditions was 0.01 mm, and the average deviation of the PSD spot position under driving conditions was 0.2 mm. The mathematical model of the PSD spot position and the algorithm of solar trajectory fast tracking are analyzed in detail, and experiments evaluate their performance. Simultaneously, the influence of vehicle-mounted SOF-FTIR on the measured spectrum is analyzed under the conditions of static vehicle, constant speed, and turning. The SOF-FTIR instrument R_SNR_ of the static on-board process is 6.597 × 10^3^:1, and that of the straight driving process is 5.703 × 10^3^:1. It is necessary to remove the measured spectrum under turning conditions to ensure the accuracy of column concentration inversion results. To solve this problem, further research will be conducted in the future on how to improve the tracking speed under turning conditions. Additionally, the application experiment of vehicle-mounted SOF-FTIR pollution gas emission flux was carried out in a tire factory in Hefei City, and the qualitative and quantitative analysis of the boundary pollution gas was realized, as well as the calculation of the flux of each component pollution gas. The results are in good agreement with the emission of pollution sources in the industrial park. The results show that the high-precision solar fast-tracking system based on PSD can meet the requirements of vehicle-mounted SOF-FTIR for solar tracking. Compared with other environmental monitoring methods, vehicle-mounted SOF-FTIR has the advantages of strong mobility and regional pollution gas emission monitoring and has a broad application prospect in pollution source control and emergency monitoring.

## Figures and Tables

**Table 1:** The main performance index of SOF-FTIR.

Atmospheric Constituents	VOCs
Spectral response range	2–16 μm
Spectral resolution	1 cm^−1^
Spectral measurement velocity	1 Spectrum/Second
Horizontal angle of solar tracker	360°
Vertical angle of solar tracker	0~89°

## Data Availability

Restrictions apply to the availability of these data.
